# Sonolucent cranial windows for bedside ultrasound identification of MRI-concordant glioblastoma recurrence during outpatient surveillance: an imaging correlation pilot study

**DOI:** 10.1007/s00701-026-06999-w

**Published:** 2026-08-08

**Authors:** Griffin Thomas, Shoaib A. Syed, Samuel Latzman, Marcio Yuri Ferreira, Seth Mejia, Artur Shlifer, Vadim Zhigin, Tamika Wong, Sanskruti Sana Bokil, Sara Massimo, Raphael Bertani, John A. Boockvar, Netanel Ben-Shalom, Randy S. D’Amico

**Affiliations:** 1 Northwell, New Hyde Park, NY USA; 2 Department of Neurosurgery, Lenox Hill Hospital, New York, NY USA; 3 New York Medical College School of Medicine, 40 Sunshine Cottage Rd, Valhalla, NY 10595 USA; 4 Division of Neurology, Department of Neurosurgery, University of São Paulo, São Paulo, SP Brazil

**Keywords:** Glioblastoma, Recurrence, Transcranioplasty ultrasound, Sonolucent cranioplasty

## Abstract

**Background and objective:**

Glioblastoma (GBM) is characterized by near-universal recurrence after surgical resection, necessitating close postoperative surveillance. Although MRI remains the gold standard for detecting tumor progression, fixed imaging intervals may delay recognition of recurrence. Sonolucent cranial implants enable transcranioplasty ultrasound (TCUS), offering a potential point-of-care adjunct for interval monitoring between scheduled MRI studies. We evaluated the feasibility of identifying sonographic findings concordant with MRI-confirmed GBM recurrence during routine outpatient follow-up.

**Methods:**

We conducted a retrospective review of prospectively registered consecutive GBM patients who underwent tumor resection with placement of a sonolucent cranial implant (ClearFit®, Longeviti Inc., Baltimore, MD) at a single tertiary center (February 2023–November 2025). Patients were included if they developed MRI-confirmed recurrence and underwent TCUS within 6 weeks before or after the recurrence-detecting MRI. TCUS examinations were performed during routine outpatient visits and interpreted by a clinician blinded to MRI findings. MRIs were independently reviewed by board-certified neuroradiologists. TCUS findings were qualitatively compared with MRI-defined recurrence.

**Results:**

Of 13 patients with sonolucent implants, 8 developed MRI-confirmed recurrence within 13 months of resection, with a range of 1 to 13 months. Four underwent TCUS within six weeks of the recurrence-detecting MRI. In all four cases, TCUS demonstrated hyperechoic abnormalities spatially concordant with MRI-defined recurrence. Examinations were safely performed (*n* = 4) in the outpatient setting.

**Conclusion:**

TCUS through sonolucent cranial implants may demonstrate concordant findings with MRI-confirmed GBM recurrence. This preliminary series supports its spatial correlation to inform future studies that evaluate TCUS as a point-of-care adjunct for interval surveillance. Prospective studies are warranted to define standardized protocols and diagnostic performance.

## Introduction

Glioblastoma (GBM) is the most common primary malignant brain tumor in adults and remains associated with poor prognosis despite advances in surgical and adjuvant therapies. Following standard-of-care treatment with maximal safe resection followed by radiotherapy and temozolomide, median progression-free survival is approximately 7 months, with median overall survival near 15 months [24, 28]. Nearly all patients ultimately experience tumor recurrence, hence the importance of close postoperative surveillance to inform timely clinical decision-making.

Magnetic resonance imaging (MRI) is the current gold standard for monitoring disease progression in patients with GBM due to its superior soft-tissue resolution and ability to characterize contrast enhancement, edema, and treatment-related effects [26]. MRI surveillance is typically performed at predefined intervals, most commonly every two to three months, in accordance with established treatment paradigms such as the Stupp protocol [28]. While commonly used, this interval-based approach creates a clinically relevant vulnerability: GBM recurrence or progression may occur between scheduled imaging studies, delaying recognition of disease progression in a tumor known for rapid and heterogeneous growth [18]. In addition, MRI-based surveillance is associated with substantial resource utilization, scheduling constraints, and patient burden, limiting flexibility in individualized follow-up strategies [26].

Recent advances in cranial reconstruction have introduced sonolucent polymethyl methacrylate (PMMA) cranial implants, which permit postoperative transcranial ultrasound imaging through the craniotomy site. Transcranioplasty ultrasound (TCUS) has been shown to be feasible for postoperative bedside assessment across a range of neurosurgical applications, including monitoring ventricular size in hydrocephalus, evaluating cerebral bypass patency, assessing mass effect, and identifying postoperative complications such as hematoma or pseudomeningocele [1, 8, 15, 23]. Elective, longitudinal TCUS monitoring has been previously documented in the context of assessing extracranial-intracranial bypass patency [7]. These prior studies support TCUS as a safe, repeatable, and cost-effective point-of-care imaging modality capable of providing longitudinal intracranial assessment outside the constraints of MRI.

Despite increasing adoption of sonolucent cranial implants and expanding use of bedside ultrasound in neurosurgery, the role of TCUS in longitudinal oncologic surveillance has not been established. Its potential for identifying glioblastoma recurrence during routine outpatient follow-up has not been systematically evaluated or correlated with MRI-confirmed progression. Moreover, even with MRI, differentiating true tumor recurrence from treatment-related changes such as pseudoprogression remains challenging, highlighting the limitations of relying on infrequent, single–time point imaging assessments alone [32].

In this study, we present the first concordance study evaluating outpatient TCUS performed through sonolucent cranial implants in effort to identify sonographic imaging signatures that appear to correlate with MRI-confirmed GBM recurrence. We do not seek to formally evaluate performance. We aim to display evidence that hyperechoic signals visualized on TCUS can be correlated to MRI-confirmed tumor recurrence.

## Methods

### Study design and ethical considerations

Following the principles outlined in the *Declaration of Helsinki* and the Preferred Reporting of Case Series in Surgery (PROCESS) 2023 guidelines, we conducted a retrospective review of consecutive cases of patients with GBM who underwent tumor resection with reconstruction using a sonolucent cranial implant at a single tertiary neurosurgical center between February 2023 to November 2025 [12]. This study was conducted under Institutional Review Board approval (Protocol #22–0873). All patients provided written informed consent for the craniotomy for brain tumor resection and intraoperative surgical reconstruction with sonolucent cranial implant placement. Consent was also obtained for outpatient TCUS evaluation, and use of de-identified clinical and imaging data for research and publication purposes.

### Patient selection and inclusion criteria

Patients were eligible for inclusion if they met all of the following criteria: (1) Age ≥ 18 years at the time of surgery; (2) Histopathologically confirmed glioblastoma; (3) Tumor resection with placement of a sonolucent polymethyl methacrylate (PMMA) cranial implant (ClearFit®, Longeviti Neuro Solutions LLC, Hunt Valley, MD, USA) which is FDA-cleared (510(k) K203349) and indicated for cranial reconstruction and postoperative ultrasound imaging. (4) Radiographic evidence of tumor recurrence identified on MRI during postoperative surveillance; and (5) Performance of transcranioplasty ultrasound (TCUS) within six weeks before or after the MRI study that identified recurrence. The temporal window was defined a priori to retrospective review in order to maximize similar findings and reduce the chance for significant additional tumor progression between the two studies. Our study aimed to capture patients in which TCUS and MRI reflected the same disease state, rather than to increase sample size. All cases meeting these criteria were included regardless of TCUS image quality. During the postoperative period, adjuvant regimens were not adjusted for the purposes of the study. Standard chemoradiotherapy (Table 1) was defined as 6 weeks of radiotherapy with concurrent temozolomide chemotherapy followed by 6 cycles of adjuvant temozolomide chemotherapy for 5 days every 28 days, as prescribed in the Stupp protocol [28].

**Table 1:** Patient characteristics at presentation

Case	Age/Sex	Tumor Location	Implant Location	Molecular Details	Extent of Initial Resection	Initial Chemo/RT Treatment Plan
1	74/F	Left temporal	Left frontotemporal	GBM, IDH-wildtype, MGMT methylated, EGFR amplified	Subtotal resection	Standard chemotherapy and RT followed by maintenance TMZ per Stupp protocol
2	79/F	Right temporal	Right temporal	GBM, IDH-wildtype, MGMT methylated	Gross total resection	Patient did not receive chemotherapy or RT
3	62/M	Middle cranial fossa and right temporal lobe	Right temporal	GBM, IDH-wildtype, MGMT methylated	Subtotal resection	Standard chemotherapy and RT followed by maintenance TMZ per Stupp protocol
4	70/M	Right temporal	Right temporal	GBM, IDH-wildtype, MGMT unmethylated, EGFR non-amplified	Gross total resection	Standard chemotherapy and RT followed by maintenance TMZ per Stupp protocol

*GBM* Glioblastoma, *RT* Radiation Therapy, *TMZ* Temozolomide, *IDH* Isocitrate Dehydrogenase, *MGMT* O^6^-methylguanine-DNA methyltransferase, *EGFR* Epidermal Growth Factor Receptor

### Sonolucent cranial implant placement protocol

At our institution, select patients undergoing resection of high-grade glioma or GBM underwent placement of a sonolucent cranial implant to facilitate postoperative TCUS surveillance. Implant placement reflected the standard operative practice of the primary surgeon performing the resection and was individualized according to patient specific considerations, including perceived infection risk, soft tissue and wound related factors, and the anticipated utility of transcranial ultrasound surveillance. The implant was placed either as a complete replacement for the autologous bone flap or as an inset within the replaced native bone flap. When used as a full replacement, the implant was contoured to the craniectomy defect and secured to the surrounding calvarium with titanium plates and screws. When inset within the native flap, the bone flap was first reaffixed, a matching segment was removed with a cutting burr, and the implant was secured within the residual bone flap using standard fixation hardware. In all cases, the cranioplasty site was irrigated with vancomycin solution, and the scalp was closed in layered fashion.

### Transcranioplasty ultrasound acquisition and interpretation

TCUS examinations were performed during routine outpatient clinic visits using a LOGIQ E10s ultrasound system (GE Healthcare, Chicago, IL, USA). Imaging was obtained using a 2–6 MHz broadband probe with a two-dimensional array and curved-linear geometry. The abdominal preset was used for grayscale structural imaging. Imaging depth was typically set between 6 and 8 cm to allow visualization of the resection cavity, surrounding parenchyma, ventricular system, and midline structures. Doppler and contrast enhanced ultrasound (CEUS) were not used, as grayscale imaging information was sufficient for the separate prospective study from which this study population was derived. The “Far” Time Gain Compensation (TGC) pre-set was used at 80% during image acquisition. Images were archived as DICOM images on an FDA and HIPAA compliant electronic binder platform (Florence Healthcare®).

Patients were imaged in the seated position without hair removal. Standard ultrasound gel was applied to the skin, and the ultrasound probe was placed directly over the implant site. Axial and sagittal sweeps were systematically conducted. The following characteristics were assessed for each patient undergoing outpatient TCUS: evaluation of the resection cavity margins, presence of abnormal blood or fluid collection, assessment intracranial anatomy including the adjacent brain parenchyma, lateral and third ventricles, falx cerebri, septum pellucidum, and choroid plexus. Tumor recurrence was defined as hyperechoic signal based on prior work characterizing high grade gliomas with distinct hyperechoic boundaries during intraoperative ultrasound [20, 21, 30].

TCUS examinations were performed and interpreted by one of two physician associates with formal TCUS training and extensive point-of-care ultrasound experience in outpatient, inpatient, and intensive care settings. Both examiners were blinded to prior and contemporaneous MRI findings at the time of acquisition and interpretation. To minimize bias from clinical information suggestive of recurrence, TCUS was performed immediately before the office visit, and the examiner was not present for the subsequent evaluation or discussion with the primary surgeon. Accordingly, TCUS was obtained during the same visit, but before MRI results were discussed with the patient.

TCUS assessments were descriptive and qualitative, focusing on identifying regions of abnormal echogenicity relative to expected postoperative anatomy, including the surgical cavity and brain parenchyma. Each TCUS exam was evaluated independently and was not compared to previous TCUS exams.

### Magnetic resonance imaging and definition of recurrence

MRI surveillance was performed using either a Siemens MAGNETOM Vida 3.0 T scanner or a Philips Ingenia Elition 3.0 T scanner. Imaging protocols included pre-contrast and post-contrast T1-weighted imaging, T2-weighted imaging, T2 FLAIR, and diffusion-weighted imaging. Gadolinium-based contrast (gadobutrol) was administered intravenously at a standard dose of 0.1 mL/kg, with post-contrast imaging acquired within a consistent time window following injection.

All MRI studies were interpreted by board-certified neuroradiologists who were blinded to TCUS findings. Each neuroradiologist was aware of the patient’s clinical history and prior head CT or MR imaging in accordance with standard institutional protocols. Tumor recurrence was defined as new or progressive foci of parenchymal or dural contrast enhancement and associated imaging features consistent with neuro-oncology imaging criteria.

### Imaging concordance assessment

For patients meeting inclusion criteria, TCUS findings were qualitatively compared with MRI-defined recurrence with respect to spatial location and relationship to the resection cavity and adjacent anatomical structures. Concordance was defined as colocalization of sonographic hyperechoic foci with MRI-detected recurrent lesions [30]. Given the exploratory nature of this proof-of-concept study with limited cohort size, formal diagnostic performance metrics were not calculated.

## Results

### Cohort overview

A total of 13 adult patients underwent brain tumor resection with placement of a sonolucent cranial implant at our institution. Each of these patients had histopathologically confirmed GBM. During postoperative surveillance, 8 of the 13 patients (62%) developed MRI-confirmed tumor recurrence ranging 1 to 13 months after surgery. Among these 8 patients, 4 met inclusion criteria for temporal concordance, having undergone TCUS within the prespecified window of six weeks before or after the MRI study that identified recurrence. The remaining four patients were excluded because they did not have TCUS acquired within the 6-week window. Key clinical and imaging characteristics for the four included patients are summarized in Tables 1 and 2.

**Table 2:** Patient characteristics at recurrence

Case	Age/Sex	Surgery-Recurrence on MRI Interval	Surgery-Recurrence on TCUS Interval	MRI-TCUS Interval	MRI Evidence of Recurrence	TCUS Evidence of Recurrence	Symptoms of Recurrence	Management Change after Recurrence	Reoperation after MRI and TCUS detection of tumor
1	74/F	78 days	110 days	+ 32	Increased contrast enhancement and T2 hyperintense regions in the left parahippocampal gyrus	Region of hyperechoic signal at the medial margin of the left temporal resection cavity correlating with T1 hyperintensity on post-contrast MR at the region of the parahippocampal gyrus	Hydrocephalus	Declined further treatment including chemotherapy or RT	No
2	79/F	131 days	145 days	+ 14	Interval development of heterogenous enhancement in the right temporal lobe suspicious for tumoral disease	Region of hyperechoic signal at the posterior-medial and posterolateral margin of the right temporal resection cavity correlating with hyperintense contrast-enhanced region at the right temporal lobe on T1 MR	Generalized lower leg weakness, emesis	Declined further treatment including chemotherapy or RT	No
3	62/M	125 days	125 days	0	Interval development of a peripheral area of enhancement in the right temporal lobe/posterior insula	Hyperechoic signal at the posterolateral margin of the resection cavity correlating with hyperintense T1 post contrast MRI signal	Decreased executive function, seizure, weakness, worsened balance and gait	Gamma Knife Radiosurgery in 5 fractions Bevacizumab therapy	No
4	70/M	337 days	349 days	+ 12	New expansile nodular lesion along the posterior lateral margin of the resection cavity	Hyperechoic signal at the right inferior temporal lobe lateral to the right lateral ventricle correlating with T1 hyperintensity	Denied new symptoms	Carboplatin therapy	Yes (gross total resection, pathology confirmed glioblastoma

*RT* Radiation Therapy

### Imaging concordance between TCUS and MRI

In all four patients, TCUS identified hyperechoic abnormalities spatially concordant with regions of MRI-defined recurrence, despite blinded interpretation. TCUS examinations were performed in the outpatient clinic setting without complications and at intervals ranging from 0 to 32 days relative to the MRI that detected recurrence. Across cases, TCUS enabled visualization of abnormalities adjacent to the resection cavity, associated parenchymal changes, and relevant intracranial landmarks, including the ventricular system and midline structures.

Two cases with representative, high-quality MRI and TCUS imaging are presented in detail to illustrate imaging concordance. All four cases demonstrated spatial concordance between TCUS findings and MRI-defined recurrence. No cases of MRI-confirmed recurrence in which TCUS was performed within 6 weeks failed to demonstrate spatially concordant hyperechoic signal.

### Illustrative case summaries

#### Illustrative case 1

**Fig. 1:**
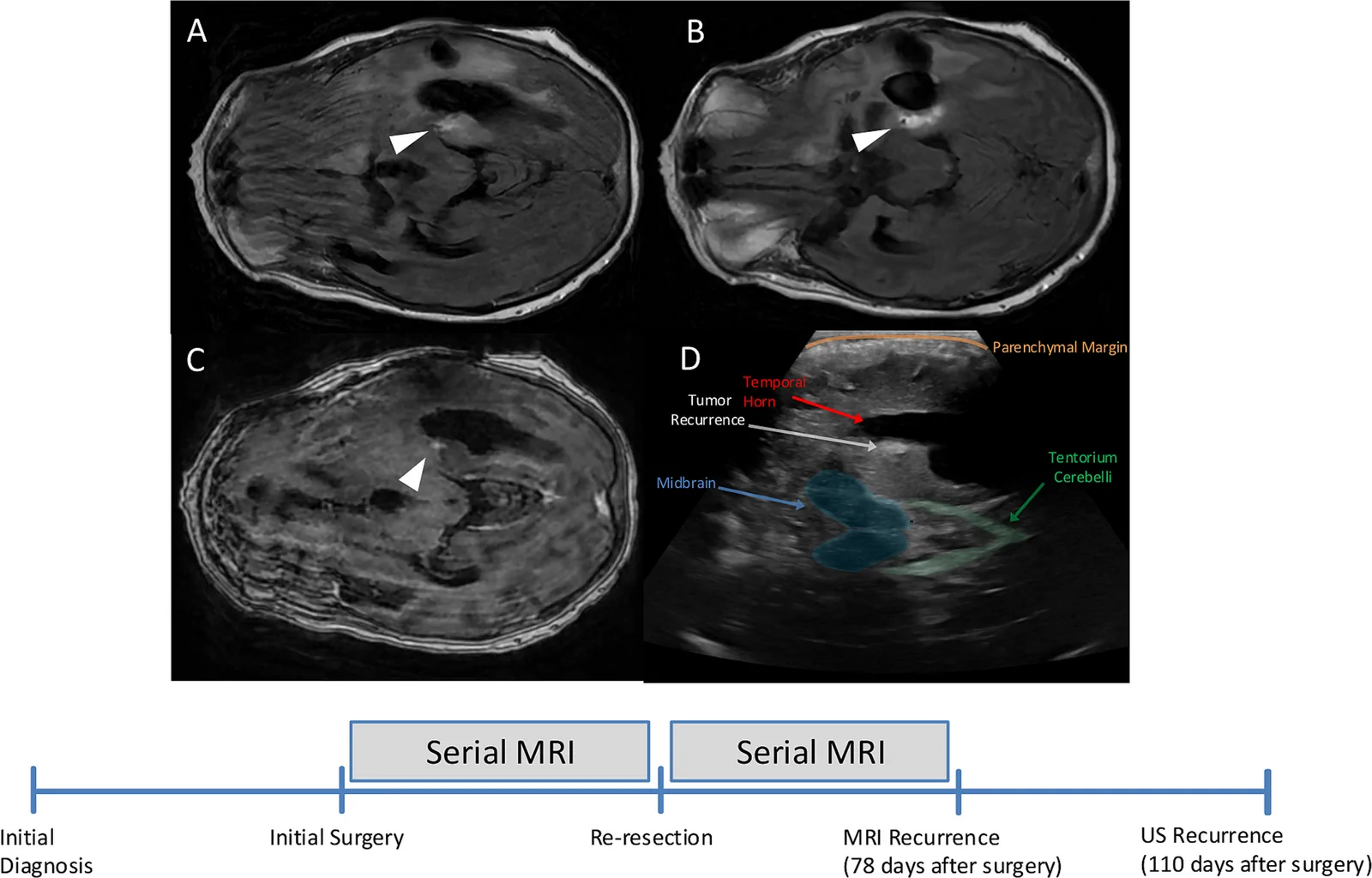
MRI and TCUS imaging for case 1. Axial T2 MRI FLAIR sequence at separate slices (**A**, **B**), and T1 post-contrast MRI (**C**) sequences obtained postoperatively demonstrated a left temporal periventricular hyperintensity and contrast enhancement near the location of the resection cavity. MRI images are rotated 90° counterclockwise to correlate with TCUS orientation. Corresponding TCUS (**D**) obtained after MRI-detected recurrence demonstrated a heterogenous oval-shaped hypoechoic abnormality with surrounding ring-shaped hyperechoic abnormality suspicious for recurrent disease abutting the lateral ventricles. These findings corresponded to the hyperintense region seen on MRI. The midbrain and tentorium cerebelli are shaded blue and green, respectively, on the TCUS image for spatial orientation. A timeline of the patient’s resection and postoperative surveillance is portrayed below the imaging, with the re-resection serving as the index operation

A 73-year-old woman underwent subtotal resection of a left temporal glioblastoma with placement of a sonolucent cranial implant. MRI surveillance demonstrated tumor recurrence 20 weeks after surgery, and the patient subsequently underwent repeat resection. Further MRI surveillance identified recurrence 11 weeks after the second resection. TCUS performed 32 days after the recurrence detecting MRI demonstrated a hyperechoic oval-shaped abnormality at the medial margin of the resection cavity, adjacent to the left temporal horn corresponding to nodular enhancement and T2 FLAIR hyperintensity on MRI, equally oval-shaped and located in the same region, as shown in Fig. 1. Following a multidisciplinary discussion, the patient elected for hospice care.

#### Illustrative case 2

**Fig. 2:**
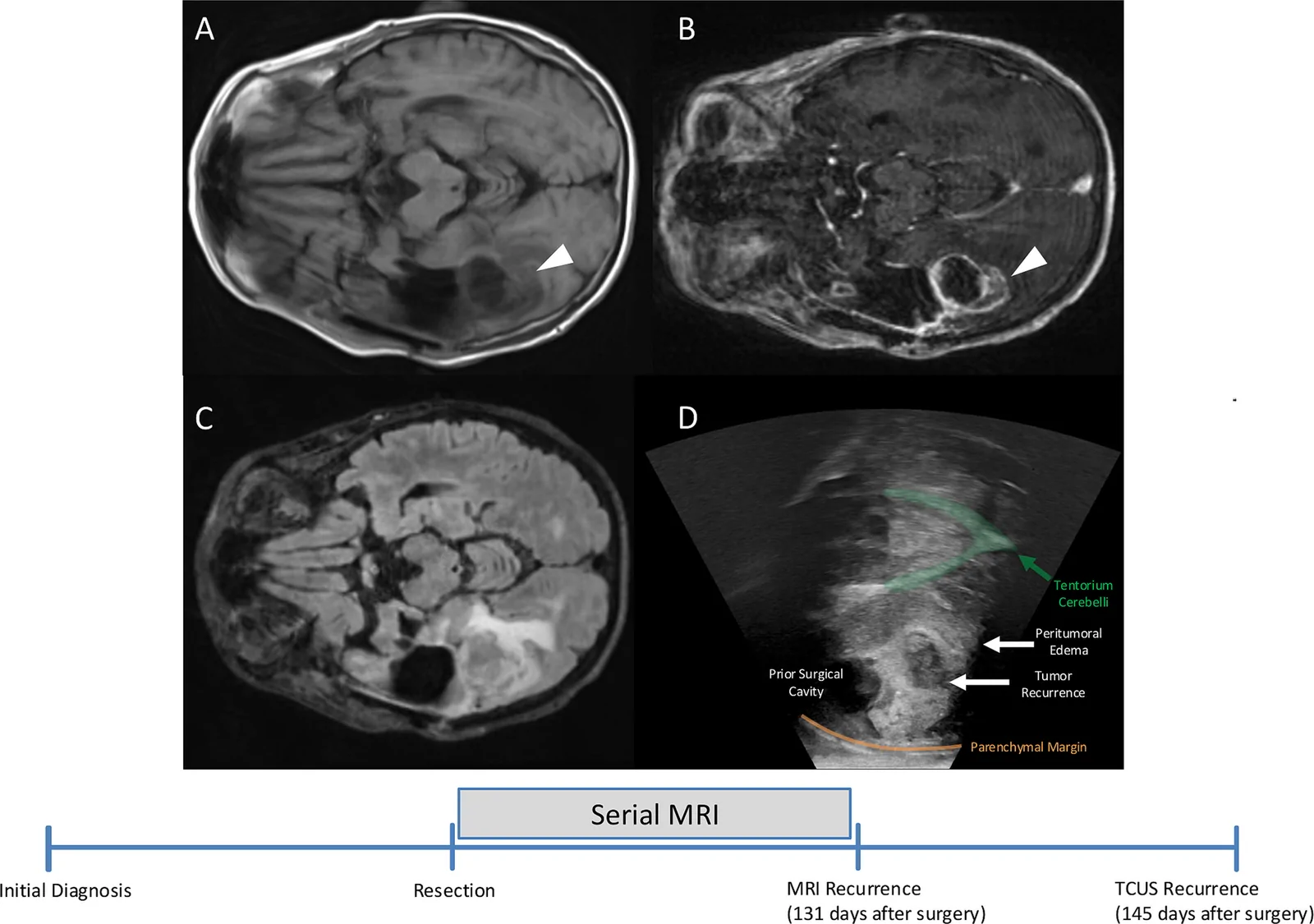
MRI and TCUS Imaging for Case 2. Axial T1 pre-contrast (**A**), post-contrast (**B**), and T2 FLAIR (**C**) MRI sequences obtained postoperatively demonstrated a contrast-enhanced hyperintense region (arrowheads) with surrounding FLAIR edema (arrow) at the posterior aspect of the right temporal resection cavity. Corresponding TCUS (**D**) obtained 14 days thereafter demonstrated a hyperechoic signal, suspicious for recurrent disease at the posterior margin of the resection cavity. The tentorium cerebelli is shaded green on the TCUS image for spatial orientation. Note, MRI images are rotated 90° counterclockwise and the TCUS image has been rotated 180° to match orientation. A timeline of the patient’s resection and postoperative surveillance is portrayed below the imaging, with the resection serving as the index operation

A 79-year-old woman underwent gross total resection of a right temporal glioblastoma with placement of a sonolucent cranial implant. MRI surveillance identified recurrence 19 weeks postoperatively. TCUS performed 14 days later demonstrated a heterogenous oval-shaped hypoechoic abnormality with surrounding ring-shaped hyperechoic abnormality at the posterior margin of the resection cavity corresponding to a newly enhancing lesion on MRI at the same location and similarly presenting central hypointensity and ring-enhancing hyperintensity (Fig. 2). The patient declined further adjuvant therapy and was lost to follow up.

#### Additional cases

Two additional patients met inclusion criteria for temporal concordance and demonstrated corresponding findings between TCUS and MRI-defined recurrence. These cases were not illustrated due to severe movement artifact on MRI. In Case 3, TCUS performed on the same day as MRI identified a hyperechoic abnormality at the posterolateral margin of the resection cavity corresponding to MRI findings. In Case 4, TCUS performed 12 days after MRI demonstrated a hyperechoic abnormality in the inferior temporal region corresponding spatially to MRI-defined recurrence. Imaging intervals, lesion locations, as well as MRI and TCUS findings for all cases are summarized in Tables 1 and 2.

### Institutional framework

**Fig. 3:**
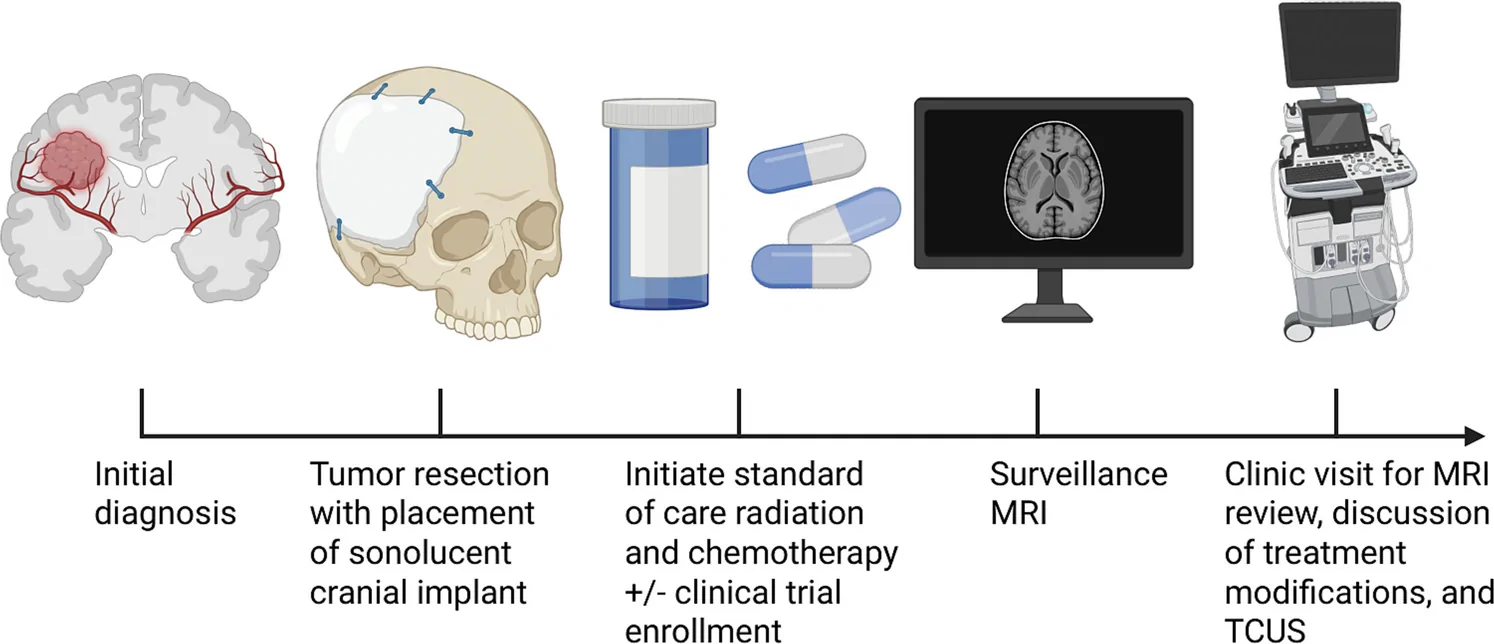
Provisional TCUS Workflow. Our institution's workflow for integrating TCUS surveillance imaging into GBM care plans, presented for hypothesis generation of more robust protocols. Created in BioRender. (2026)

Across all four cases, TCUS identified sonographic abnormalities corresponding to MRI-confirmed glioblastoma recurrence. These observations highlight the ability of outpatient TCUS to identify imaging signatures associated with tumor recurrence but do not assess diagnostic accuracy or distinguish tumor from treatment-related effects. To facilitate hypothesis generation for future studies that evaluate TCUS-MRI quantitatively and distinguish tumor from treatment related effects we present our institution's protocol for introducing TCUS into GBM surveillance (Fig. 3). In this framework, TCUS functions as a low-threshold, repeatable assessment tool performed during routine clinic visits. Abnormal or evolving sonographic findings would prompt expedited MRI and multidisciplinary review, while stable examinations could support continued observation until scheduled imaging. Such an approach aligns with prior recommendations emphasizing TCUS as an adjunctive, rather than standalone, imaging modality.

## Discussion

In this retrospective imaging concordance study, we demonstrate that TCUS performed through sonolucent cranial implants can identify hyperechoic sonographic abnormalities spatially concordant with MRI-confirmed glioblastoma recurrence during routine outpatient follow-up. Hyperechoic signal was designated to represent tumor recurrence based on prior work characterizing high grade gliomas with distinct hyperechoic boundaries during intraoperative ultrasound [20, 21, 30]. Across all four cases, TCUS identified focal hyperechoic regions of likely tumor recurrence near the MRI-confirmed site of recurrence.

These four cases present the first correlation of MRI-confirmed tumor recurrence with TCUS signals that suggest tumor recurrence. While this hyperechoic signal alone is relatively non-specific, spatially concordant MRI hyperintensity and identification of adjacent intracranial anatomy on both MRI and TCUS (Figs. 1 and 2) characterize the hyperechoic signal to be suggestive of tumor recurrence. Colocalization of hyperechoic regions with contrast enhancing regions on MRI was a crucial finding to support differentiation of edema from potential tumor recurrence, both of which produce hyperechoic signal on ultrasound [31]. In two of the four cases, TCUS and MRI findings informed additional treatment (Table 2). While case 2 did not receive chemoradiotherapy, we believe this strengthens the findings in this study by removing the cofounding impact of treatment-related changes on TCUS interpretation. These findings establish imaging concordance of clinic-based TCUS as a point-of-care adjunct to standard of care MRI for interval surveillance in patients with GBM and provide a scientific basis for future prospective studies.

The clinical challenge motivating this work is not the inadequacy of MRI, but rather the rigidity of interval-based surveillance in a disease characterized by rapid and heterogeneous progression. Standard follow-up imaging schedules, typically every two to three months as established in the Stupp protocol, may allow clinically meaningful disease evolution to occur between scans [28]. TCUS offers a practical mechanism to increase surveillance frequency without imposing the logistical and resource constraints associated with additional MRI examinations [26]. In this context, TCUS could eventually serve as a surveillance trigger rather than a diagnostic replacement, prioritizing early identification of interval change and timely escalation to conventional imaging when warranted. TCUS has been demonstrated as cost-effective relative to MRI or CT imaging while comparably revealing intracranial structures. While these savings are expected to remain for TCUS monitoring of GBM, formal cost analysis was not conducted in this study and should be evaluated to establish clinical utility [3, 16, 22].

Importantly, TCUS shares interpretive limitations with other postoperative imaging modalities. Distinguishing tumor progression from treatment-related effects or peritumoral edema remains a fundamental challenge in GBM surveillance and is not unique to ultrasound-based imaging [10]. On conventional grayscale ultrasound, echogenic abnormalities reflect alterations in tissue microstructure and acoustic interfaces rather than tumor-specific biology, resulting in overlap between infiltrative tumor, vasogenic edema, gliosis, calcification, and radiation-related change. Prior intraoperative and bedside ultrasound studies have consistently noted this limitation, emphasizing ultrasound’s strength in defining lesion geometry and interval change rather than definitive tissue characterization [19, 30, 31]. Ultrasound imaging also faces the unique challenge of being operator dependent, introducing potential inter and intra-patient variability across their clinical course. TCUS specifically is limited by the sonolucent window created by the PMMA implant, preventing detection of distant tumor recurrence. Considering these limitations, it must be emphasized that while TCUS findings are suggestive of tumor recurrence, vasogenic edema, gliosis, or calcification cannot be ruled out and further studies are needed to definitively define the presence of tumor recurrence on TCUS.

Moreover, even MRI faces diagnostic ambiguity in differentiating true tumor recurrence from pseudoprogression or radiation-related injury, particularly in the early post-treatment period [10]. Accordingly, the findings presented here should not be interpreted as evidence that TCUS can independently distinguish recurrence from treatment-related effects, but rather as support for its role in detecting interval deviation from an individual patient’s postoperative baseline.

The concordance of TCUS to MRI demonstrated in this series builds upon prior work establishing TCUS as a safe and practical adjunct for postoperative assessment. Ultrasound through sonolucent cranial implants has been shown to reliably visualize intracranial structures for monitoring ventricular size, cerebral bypass patency, mass effect, and postoperative complications such as hematoma and pseudomeningocele [1, 7, 8, 15]. PMMA cranioplasty implants have also shown similar complication profiles to autologous bone cranioplasties [9, 25]. A systematic review evaluating autologous cranioplasties versus alloplastic materials found higher bone resorption rates and removal rates for alloplastic materials [29]. Further, PMMA is water/soft tissue equivalent for megavolatage photons, and thus should not meaningfully perturb the dose of radiation delivered to patients in the Stupp protocol [2]. Our findings extend these applications into the oncologic surveillance domain, demonstrating that TCUS can be integrated into outpatient workflows to monitor imaging changes that may be associated with tumor recurrence.

Although formal cost-effectiveness was not evaluated in this study, TCUS represents a low-resource, point-of-care imaging modality that can be performed during routine outpatient visits without the infrastructure, scheduling demands, or patient burden associated with MRI. The potential impact of TCUS on imaging utilization, workflow efficiency, and healthcare resource allocation warrants formal evaluation in prospective studies.

Advances in ultrasound technology may further expand the applications of TCUS in neuro-oncologic care and surveillance. TCUS mediated blood brain barrier disruption may allow more effective drug delivery [6, 11, 27]. Doppler interrogation can provide complementary information regarding vascular patterns and flow, while contrast-enhanced ultrasound (CEUS) has demonstrated improved delineation of tumor perfusion and residual disease in intraoperative and experimental glioma imaging settings [20, 22, 30]. These techniques offer signals beyond grayscale US and may improve tissue characterization. However, whether Doppler or CEUS can reliably differentiate recurrent tumor from treatment-related effects or pseudoprogression in the outpatient surveillance setting remains insufficiently studied and requires prospective validation. Incorporation of multiparametric ultrasound techniques into standardized TCUS protocols represents an important area for future investigation.

Successful integration of TCUS into longitudinal GBM surveillance requires attention to several practical considerations. Reliable acoustic access to the region of interest depends on implant positioning and reconstruction strategy, emphasizing the importance of surgical planning when ultrasound-based follow-up is anticipated. Reproducible acquisition and interpretation necessitate operator training and standardized imaging protocols to ensure consistency across visits and readers. Furthermore, incorporating our proposed TCUS framework into routine follow-up workflows requires coordination among surgical, oncologic, and radiologic teams to ensure that concerning sonographic findings prompt appropriate escalation rather than isolated interpretation. Prospective studies are warranted to add standardized ultrasound operator guidelines to the framework that outline standard imaging techniques and structures to visualize when performing TCUS in this patient population.

### Limitations

Despite the practicality of TCUS in this study, several limitations exist. The sample size is small and derived from a single institution, precluding assessment of performance such as sensitivity or specificity. The size was further limited by the lack of a consistent protocol for TCUS relative to MRI acquisition, as only four of the eight patients with tumor recurrence had MRI and TCUS within a timeframe that would reasonably allow both imaging modalities to capture a similar disease state. Implementation of consistent protocols to ensure TCUS and MR imaging is obtained within the same temporal window will be necessary for feasibility studies and formal evaluation of TCUS detection of tumor recurrence. The variation in the molecular profile of the GBM in each patient is also a limitation of this study, as these variables are independent prognostic factors [4, 17, 33]. The use of grayscale ultrasound alone reduces the precision with which potential recurrence was detected, and future studies should incorporate doppler ultrasound and CEUS, which have been shown to improve delineation [1, 5, 15, 20, 30]. This study was limited to grayscale by its retrospective nature. The separate, prospective study from which this patient population was derived relies on general qualitative observations and thus does not obtain doppler or CEUS imaging. Thus, future studies should utilize doppler and/or CEUS to enhance B-mode findings here and further establish MRI-TCUS concordance.

The same two physician associates conducted TCUS examinations longitudinally. With only two examiners, the lack of inter-rater reliability limits this study, especially when considering the inherently subjective nature of grayscale ultrasound image interpretation. TCUS interpretation was performed by experienced operators, and performance may differ in broader practice settings without standardized training. Detecting tumor recurrence on TCUS remains complicated by pseudoprogression, radiation necrosis, or gliosis, all of which may produce unique sonographic signatures.

The retrospective design and reliance on qualitative imaging concordance also limits generalizability. Future studies will incorporate quantitative co-registration of TCUS and MRI findings through centroid distance and Dice overlap assessments [13, 14].

These limitations are inherent to early stage investigations and highlight the need for prospective, multicenter studies with harmonized acquisition protocols, blinded interpretation, and correlation with clinical outcomes.

## Conclusion

This proof-of-concept series aimed to demonstrate that TCUS performed through sonolucent cranial implants can identify hyperechoic sonographic abnormalities spatially concordant with MRI-confirmed glioblastoma recurrence during outpatient follow-up. These findings warrant future studies to establish the clinical utility of TCUS surveillance by defining performance metrics and hyperechoic tissue characterization.

## Data Availability

No datasets were generated or analysed during the current study.

## Abbreviations

GBM: Glioblastoma
MGMT: O^6^-methylguanine-DNA methyltransferase
PMMA: Poly(methyl-methacrylate)
TCUS: Transcranioplasty Ultrasound
